# Ohio Moves for State License

**Published:** 1892-01

**Authors:** 


### OHIO MOVES FOR STATE LICENSE.

On Thursday, December 3, 1891, a meeting of the representatives of the several medical colleges in the State of Ohio was convened at the Neil House, at Columbus, upon the call of Dr. Chas. A. L. Reed, of Cincinnati.

The object of this meeting was to consider the propriety of asking the State Legislature, at its approaching session, to pass a law providing for a State Medical Examining and Licensing Board, and further to seek the coöperation of the medical societies and physicians throughout the State in bringing about such desirable legislation.

One delegate from each medical college was invited to attend the conference, and it is understood that every college was represented at the meeting. In view of the fact that there had been some recent exposures in regard to the selling of diplomas in that State, this seems to be an opportune moment to unite the profession and the colleges, as well as all progressive and law-abiding citizens, in an effort to render such nefarious schemes impossible in the future.

We wish our neighbors and *confrères* in Ohio a full measure of success in their worthy undertaking, but we warn them that they will meet opposition from every possible source—regulars, irregulars, and quacks—so they must be on the alert in order to overwhelm the unscrupulous combinations of designing men.[1]

Chloralamid in Epilepsy.— At the October meeting of the New York Academy of Medicine, Dr. Chas. L. Dana, Professor of Diseases of the Mind and Nervous System, New York Post-Graduate Medical School, in speaking of the symptomatic treatment of epilepsy, refers to chloralamid as follows: "The most valuable adjuvant was hydrate of chloral, but I have found a new drug in chloralamid, which does all the good ascribed to the former drug, without affecting the heart or circlation."

1. Since the foregoing was written, we have received the minutes of the meeting referred to, and publish them in another column.

